# Umbilical Cord Blood Sampling for Newborn Screening of Pompe Disease and the Detection of a Novel Pathogenic Variant and Pseudodeficiency Variants in an Asian Population

**DOI:** 10.3390/ijns11030074

**Published:** 2025-09-03

**Authors:** Fook-Choe Cheah, Sharifah Azween Syed Omar, Jasmine Lee, Zheng Jiet Ang, Anu Ratha Gopal, Wan Nurulhuda Wan Md Zin, Beng Kwang Ng, Shu-Chuan Chiang, Yin-Hsiu Chien

**Affiliations:** 1 Department of Paediatrics, Faculty of Medicine, Universiti Kebangsaan Malaysia, Kuala Lumpur 56000, Malaysia; shazween@hctm.ukm.edu.my (S.A.S.O.); leejsmn@ukm.edu.my (J.L.); zhengjieta@gmail.com (Z.J.A.); anugpl@yahoo.com (A.R.G.); wannurul@hctm.ukm.edu.my (W.N.W.M.Z.); 2 Department of Obstetrics and Gynaecology, Faculty of Medicine, Universiti Kebangsaan Malaysia, Kuala Lumpur 56000, Malaysia; nbk9955@hctm.ukm.edu.my; 3 Department of Medical Genetics, National Taiwan University Hospital, Taipei City 10041, Taiwan; chiangsc@ntu.edu.tw (S.-C.C.); chienyh@ntu.edu.tw (Y.-H.C.)

**Keywords:** infantile-onset Pompe disease, late-onset Pompe disease, acid alpha-glucosidase, umbilical cord blood, newborn screening, pseudodeficiency allele, glycogen storage disease, lysosome, enzyme replacement therapy, mass spectrometry

## Abstract

Pompe disease is an autosomal recessive metabolic disorder caused by acid alpha-glucosidase (GAA) deficiency. The use of umbilical cord blood (UCB) for newborn screening (NBS) of Pompe disease, compared to heel-prick sampling, has not been widely studied. This study compared GAA activity in UCB from term newborns with peripheral or heel-prick blood samples obtained on days 1, 2, and 3 after birth. Enzyme assays were performed using UPLC-MS/MS. Sanger sequencing was conducted in infants with low GAA activity to identify pathogenic variants. Among 4091 UCB samples analyzed over 18 months, the mean GAA activity was 10.04 ± 5.95 μM/h, higher in females than males [Median (IQR): 9.83 (5.45) vs. 9.08 (4.97) μM/h, respectively, *p* < 0.001], and similar across ethnicities. GAA levels in UCB and Day 3 heel-prick samples were comparable. A GAA cut-off value of 1.54 μM/h (0.1% of study population) identified one infant (0.024% prevalence) with a novel bi-allelic variant—c.2005_2010del (p.Pro669_Phe670del) and c.1123C>T (p.Arg375Cys), and 12 infants with non-pathogenic pseudodeficiency alleles. This study supports GAA measurement in UCB as a viable alternative for NBS, with enzyme activity remaining stable for up to 72 h post-collection. Larger-scale multicenter nationwide studies are warranted to confirm this prevalence in our population.

## 1. Introduction

Pompe disease is a rare autosomal recessive inherited inborn error of metabolism affecting one in 40,000 individuals worldwide [1]. It is caused by a deficiency in acid alpha-glucosidase (GAA), an enzyme integral for breakdown of glycogen polymers into glucose within lysosomes [2,3]. Accumulation of glycogen in various tissues results in pathological manifestations of progressive muscle weakness [4]. Pompe disease has a spectrum ranging from the severe form, infantile-onset Pompe disease (IOPD), to the relatively mild to moderate disease in adulthood [5]. The IOPD is associated with an average mortality at 8.7 months old if not treated early [5]. Pompe disease can be detected by screening newborn infants for deficiency of the GAA enzyme [6] and confirmed through genetic testing [7]. Preventive treatment is available with early initiation of enzyme replacement therapy (ERT) that markedly improves patient outcomes [8].

Current NBS programs predominantly detect Pompe disease by measuring GAA in dried blood spots (DBS) collected from heel-prick sampling between 24 and 72 h after birth, a method that has been proven to be an accurate first-line screening tool for Pompe disease [9,10]. For individuals with low GAA enzyme activity, confirmatory testing is typically conducted through repeat enzyme assays and molecular genetic analyses. In 1980, Malaysia began conducting NBS for glucose-6-phosphate dehydrogenase deficiency using UCB, followed later by congenital hypothyroidism [11]. However, the expansion of the current NBS to include various inborn errors of metabolism through DBS testing is not yet implemented. Certain barriers include the logistics involved with DBS collection, early discharge of newborn infants from hospital, and the lack of validation data of enzyme levels in UCB versus peripheral blood samples [12].

It has been shown that it is possible to screen for inherited metabolic conditions such as Pompe disease by determining the concentration of the enzyme in plasma as well as from DBS [13]. Since there is no established range of GAA enzyme level defined for Pompe disease based on UCB, this study aims to validate the enzyme activity in UCB compared to heel-prick blood samples on DBS. The recommended heel-prick sample collection to screen for Pompe disease as part of the newborn screening program in Taiwan using the DBS method is 2–3 days after birth, but mothers and newborns are often discharged even within 24 h after delivery in many countries including Malaysia. Therefore, utilizing UCB samples which are readily available offers a pragmatic alternative to expand the NBS in countries that have been using this as an existing approach. The use of UCB in NBS is particularly favorable in countries with a high hospital turnover rate or lacking in a community healthcare service to conduct heel-prick blood sampling.

We analyzed more than 4000 UCB samples from term newborn infants in a Malaysian hospital and the results indicate that UCB on DBS is comparable to heel-prick blood GAA at Day 3 after birth of a Taiwanese newborn population using a similar tandem mass spectrometry facility. We also detected a case with novel mutation and several pseudodeficiency alleles of the GAA gene in our population.

## 2. Materials and Methods

### 2.1. Data Collection

This prospective, observational study was conducted at the Hospital Canselor Tuanku Muhriz (HCTM), Kuala Lumpur, Malaysia, a teaching hospital of Universiti Kebangsaan Malaysia (UKM), with an estimated 4000 births per year. The study was approved by the UKM Research Ethics Committee (project approval reference code—UKM PPI/111/8/JEP-2021-471). It was conducted over a period of 18 months, from November 2021 to May 2023. Sampling was performed by purposive method and term infants were enrolled after written parental consent.

### 2.2. UCB Versus Peripheral Blood Sampling for GAA Levels

Our main objective was to determine the range of GAA levels in the UCB of newborn infants. It was estimated that GAA levels from at least 4000 term infants born over a one-year period in this hospital will be ideal to create a representative curve to establish the distribution up to the 99th percentile of the GAA levels from this population with diverse ethnicities, the majority of whom are Malays, Chinese, and Indians.

Our second objective was to determine if UCB was comparable with peripheral venous blood GAA levels of infants shortly after birth. The idea behind this was that if the UCB sample was missed, inadequate, or hemolyzed, then a venous blood sample may be a suitable replacement. For this, a subset of 100 term infants who were otherwise healthy but were not discharged within the first 24 h after birth was randomly sampled after obtaining parental consent. The minimum sample size needed for the intraclass correlation coefficient (ICC) analysis was computed using R Package ‘ICC.Sample.Size’, which was based on a method by Zou (2012) [14].

Our third major objective was to compare GAA levels in UCB from all the term infants in this study against the historical cohorts of GAA levels in DBS from heel-prick samples obtained at Day 2 and Day 3 from the newborn population of Taiwan, assayed in the National Taiwan University Hospital (NTUH) using the same analytical method.

**Figure 1:**
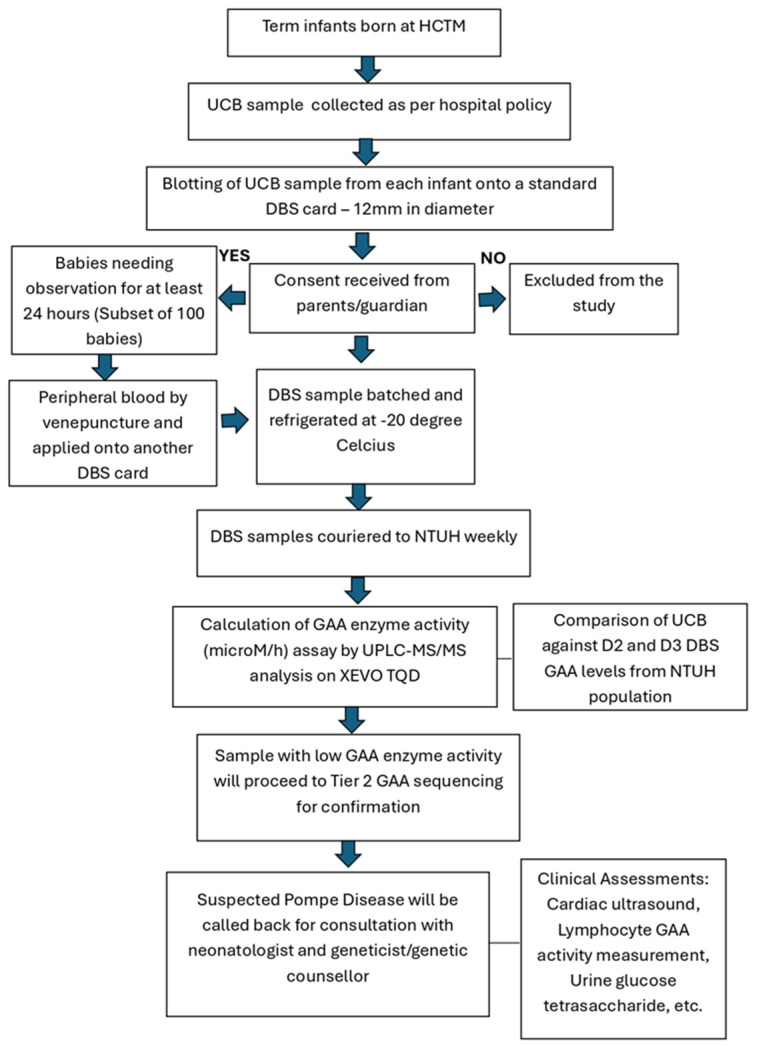
The collection of UCB and peripheral blood samples from term infants for GAA levels.

UCB samples were obtained as per the current standard of practice in this hospital as shown in Figure 1. The UCB samples, on average about two mL each, were routinely collected in EDTA tubes (BD Diagnostics, Franklin Lakes, NJ, USA). Approximately 50 µL of this blood sample for each baby were spotted evenly on NTUH inhouse DBS filter paper card within the pre-printed circle of 12 mm in diameter. For the subset of 100 infants, each would have paired collected samples of UCB and Day 1 peripheral blood on separate DBS cards. The DBS cards were kept refrigerated (−20 °C) before they were couriered in batches on a weekly basis to NTUH for assay.

In aligning with good clinical practice, infants with low GAA activity samples, GAA gene sequencing was conducted. For any case with 2 pathogenic or likely pathogenic variants identified, further diagnostic evaluation was conducted to establish a definitive diagnosis. In such a situation, we provided a call-back for consultation with the attending neonatologist and geneticist/genetic counsellor in this hospital. The recommended diagnostic evaluation included a complete cardiac and physical examination, repeat DBS GAA measurement, and/or lymphocyte GAA activity measurement, urine glucose tetrasaccharide (Glc4) measurement, and CK measurement. For these cases, genetic counselling for both parents’ GAA carrier status determination and the sibling’s status would also be offered.

### 2.3. UCB Storage Time on GAA Levels

We also sought to determine the versatility of UCB samples kept in EDTA tubes with storage at 4 °C in the refrigerator over time before blotted to make DBS, if the levels of GAA change significantly. An aliquot of UCB samples were taken after 24 h, 48 h, 72 h, and 96 h storage, respectively, to be blotted onto the DBS card and these respective timed samples were compared against the level of the UCB when freshly blotted (0 h). In a further smaller subset of these samples, we also compared the GAA levels from the same UCB sample on DBS dried in less than 6 h (the usual drying period) against 24 h (overnight). The minimum sample size for each of these subgroups was 30 samples to ensure the sampling distribution approximates the Gaussian distribution according to the central limit theorem [15].

### 2.4. Laboratory Assay of DBS for GAA Levels

The DBS were sent for assay at the Newborn Screening Center of the NTUH that performs routine screening for one-third of the newborn infants in Taiwan, which approximates 70,000 births annually. The center has performed regular Pompe disease screening since 2008. The GAA enzyme activities were measured using PerkinElmer NeoLSD (Turku, Finland) as described [16]. In brief, 3 mm filter paper samples were punched out from the respective DBS cards using a Wallac DBS Puncher. These samples were transferred into wells of a 96-well microtiter plate containing lysosomal storage disease (LSD) quadruplex assay cocktail to screen for Pompe, Fabry, Gaucher, and MPS I diseases. For this study, the DBS samples will be analyzed for Pompe disease only. However, running the quadruplex assay for the other LSDs concurrently is integral for quality control and verification of the analytical output. Plates were sealed with aluminium sealing film for 16 h incubation at 37 °C with orbital shaking. After overnight incubation, the enzyme reaction was quenched with 100 µL of 1:1 methanol/ethyl acetate solution. The products and internal standards were separated from the buffer by liquid-liquid extraction using 400 µL of ethyl acetate and 200 µL of purified water for the assay. Aliquots 200 µL of the ethyl acetate phase from the wells were evaporated and reconstituted in 45% acetonitrile with 0.1% formic acid for UPLC-MS/MS analysis.

### 2.5. UPLC-MS/MS Analysis

UPLC-MS/MS was performed on a XEVO TQD triple-quad mass spectrometer (Waters, Milford, MA, USA) in the positive ion mode. Aliquots of the samples (5 µL) were injected into an analytical column (ACQUITY UPLC CSH C18, Waters, Milford, MA, USA; 2.1 × 50 mm, 1.7 μm) with a gradient separation by mobile phase A (30% acetonitrile/70% water with 0.1% formic acid) and mobile phase B (50% acetonitrile/50% isopropanol with 0.1% formic acid) at a flow rate of 0.8 mL/min at 55 °C under the following gradient conditions: linear gradient from 1% to 70% B from 0 to 1.0 min; linear gradient from 70% to 75% B from 1.0 to 1.5 min; decreased to 1% B and re-equilibrated for 0.5 min. Data were collected during 1.6 min of sample infusion. The total running time for one plate was approximately 3 h. The enzyme activity was calculated in µM/h from the ratio of isotopic-substituted enzymatic product to internal standards.

In NTUH, the GAA levels taken as cut-off values to flag as abnormal are based on validation results published previously [17], whereby the deficient level cut-off is 1.2 µM, equivalent to GAA levels of 0.1 percentile of the population and the critically low cut-off is 0.6 µM, equivalent to GAA levels of 0.01 percentile of the population. As this study was conducted in a different population, different cut-off values were explored based on the assay values gathered.

### 2.6. GAA Gene Sequencing

Molecular genetic analysis of the GAA gene was conducted based on the Sanger sequencing method to detect pathogenic variants in exons and exon–intron junctions of the gene after genomic DNA was extracted. Measurement of the purity and concentration of extracted DNA was performed by using a NanoDrop Spectrophotometer. DNA amplification to validate the variants in the GAA gene (NM_000152.4) was carried out through polymerase chain reaction (PCR). This was performed using specific forward and reverse primers flanking the 20 exons, including the splice sites. BigDye Terminator Cycle Sequencing V3.1 chemistry (Applied Biosystems, Foster City, CA, USA) was used to perform bi-directional DNA sequencing. Analysis of DNA sequencing data was conducted using SeqScape software version 3.0 (Applied Biosystems, Waltham, MA, USA). Identified variants were referenced with the Human Gene Mutation Database (HGMD) to evaluate their significance and to determine whether the variants had been previously reported. Parental testing was performed to evaluate variant segregation and to substantiate the diagnosis.

### 2.7. Statistical Analyses

To determine the relationship/correlation of GAA in UCB with peripheral blood samples on Day 1 and NTUH historical data on heel-prick DBS collected on Day 2–3, the chosen analysis for this purpose relied on two cascading steps. The paired data in each storage and drying subgroups were first tested using paired t-test analysis via SPSS version 21 software (IBM Corp, Armonk, NY, USA). If there was evidence of non-normality of the paired-data differences via Kolmogorov–Smirnov test, Wilcoxon signed rank test was used instead. For either test, if the difference was found to be not statistically significant, the analysis would proceed with equivalence testing to evaluate the agreement of the measurements. The equivalence testing was performed using the two-one-sided-test (TOST) method, whereby the null-hypothesis of non-equivalence was divided into two one-sided null hypotheses [18] and the TOSTER Package via R version 4.2.2 software was used [19]. The choice of the equivalence bound was based on the estimated minimal important difference of the GAA enzyme level. There is measurement agreement if both TOST equivalence bounds are statistically significant (*p*-value < 0.05) [20]. Weighted analysis was conducted to compensate for the proportion differences in patient ethnic composition in this study compared to the actual ethnic composition of the Malaysian population. This adjustment was intended to better generalize if present, any ethnicity-related differences in enzyme levels. The weighted analyses used the general linear model in SPSS, whereby the sample weight was based on the post-stratification weight and the estimation of error was based on sampling with a replacement design.

The agreement between the measurements is further illustrated using the Bland–Altman plot using MedCalc version 14 software (MedCalc Software Ltd., Ostend, Belgium). The 95% CI of mean difference and 95% CI of limits of agreement are illustrated in the plot. All data were analysed using IBM SPSS version 21 software (IBM Corp, Armonk, NY, USA) unless otherwise specified.

## 3. Results

### 3.1. Descriptive Analyses of UCB GAA Levels

**Figure 2:**
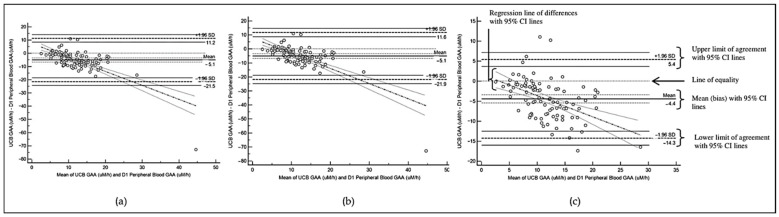
Bland–Altman Plot for comparison of GAA levels between UCB and D1 peripheral blood based on (**a**) Dataset A; (**b**) Dataset B; (**c**) Dataset C.

UCB samples were collected from 4091 newborn infants from November 2021 to May 2023 at HCTM. The number of samples used for analyses of GAA levels were divided to three datasets (A, B, C) based on exclusion of some samples for the reasons stated in each of the categories (Figure S1). Results are presented in such a way as they represent real-world data obtained in a normal clinical setting. Descriptive analyses of the cord blood GAA are shown in Table 1 and Figure 2.

The mean GAA enzyme level in UCB (Dataset A) was approximately 10.04 µM/h (SD = 5.95), while the median GAA level was 9.43 µM/h (IQR = 5.25) Table 1. The distribution of GAA levels demonstrated an improvement in normality after the removal of extreme outliers (Dataset C: skewness = 0.76, kurtosis = 0.81) (Figure S2). Using the datasets obtained, we classified GAA enzyme activity based on two different definitions for the cut-off levels to determine the risk for Pompe disease. The classification reported by Burlina et al. [21] states that a borderline value of GAA is when the level is between “0.2 × Median value of GAA enzyme activity” and “0.25 × Median value of GAA enzyme activity” of the reference dataset. The value of GAA for high risk of Pompe disease is below “0.2 × Median value of GAA enzyme activity” of the referred dataset. The NTUH classification for high possibility of Pompe disease is determined at a cut-off value below 0.1 percentile of GAA enzyme activity in the reference dataset. Based on these definitions, the GAA values are compared and shown in Table 2.

Based on the Burlina et al. classification, the percentage distribution of normal and at-risk newborns across the different categories are: 99.2% of infants have normal GAA levels, 0.5% have borderline GAA levels, and 0.3% (*n* = 10) are classified as having high-risk for Pompe. In comparison, NTUH classification of high possibility for Pompe disease with GAA cut-off value of 0.1% of the population average means only four infants are at risk (Table S1).

### 3.2. Demographic Factors Associated with UCB GAA Levels

We also wanted to determine if GAA levels are related to sex, birth weight, and ethnicity. Details of this study population demographic characteristics are shown in Table S2. For birth weight, the WHO classification is used: (i) macrosomia (>4.00 kg); (ii) normal (2.50 kg to 4.00 kg); (iii) low birth weight [LBW] (>1.50 kg and <2.50 kg); (iv) very low birth weight [VLBW] (>1.00 kg and ≤1.50 kg); and (v) extremely low birth weight [ELBW] (≤1.00 kg). Ethnicity was based on the maternal registered ethnic group in the hospital records.

Female infants have significantly higher UCB GAA compared to male infants. The relationship between UCB GAA and birth weight showed a very small sized statistically significant positive correlation. These findings were consistent across the three datasets. With regards to the birth weight, based on the post hoc test performed on the Dataset C, the significant difference was primarily due to a significantly higher value of cord blood GAA for normal birth weight compared to the LBW category. However, UCB GAA levels were not different across the different ethnicities (Table S2).

### 3.3. UCB and Peripheral Venous Blood GAA Levels

In this section of the study, paired UCB and peripheral venous blood obtained within 24 h after birth from 108 infants were available. There are three datasets (A, B, C) used in this section for data analyses with the reasons for each category specified in Figure S3.

The descriptive analyses of the GAA value differences between cord blood and peripheral blood are summarized in Table 3. There are statistically significant differences between the UCB and D1 peripheral venous blood GAA levels, which are consistent across the three datasets. The Bland–Altman plots (as shown in Figure 2) demonstrate consistently negative bias between UCB and D1 peripheral blood GAA values, whereby the UCB GAA values are consistently lower in all the datasets.

As the UCB are significantly lower than Day 1 peripheral blood GAA levels, we attempt to produce a multiple linear equation model using dataset C with normally distributed data to link UCB and peripheral blood GAA values. The multiple linear regression construct has peripheral blood GAA as the dependent variable and cord blood GAA as the main independent variable, controlling for birth weight. For newborn infants with birth weight ranging between 2.5 and 4.0 kg, the best equation to link Day 1 peripheral blood GAA and UCB GAA is:

UCB GAA, μM/h = 0.421 (Peripheral blood GAA value taken within 1 day after birth) + 3.557

### 3.4. Comparison of GAA Levels in UCB with Heel-Prick Blood Samples Taken at Day 2 and Day 3

The results in this section were based on analyses of UCB GAA levels from Dataset C (*n* = 3980) (Table 1) of infants born in HCTM when compared with the heel-prick blood sample results obtained at Day 2 (*n* = 3796) and Day 3 (*n* = 5332) from DBS of Taiwanese newborn infants screened at the NTUH. The results indicate mean GAA in UCB (9.96 SD 4.05) is significantly lower than Day 2 heel-prick blood sample (11.33 SD 4.53), but UCB GAA is not significantly different from heel-prick blood sample taken at Day 3 (10.18 SD 4.23).

### 3.5. UCB Sample Storage and DBS Overnight Drying on GAA Levels

In determining the best practice for UCB sample collection process before blotting onto DBS card and drying it for GAA assay, we compared the effects of the different lengths of storage and drying time on the activity level of the GAA enzyme.

The UCB samples were kept in the refrigerator at 4 °C assigned to four different groups with the storage duration of 24 h, 48 h, 72 h and 96 h. Another group was added using residual UCB samples that had been kept over the weekend (48-h) that were then stored for another 48-h (total duration 96 h) before blotting onto the DBS card. For internal validity purposes, the same UCB of a subject was used to compare GAA levels over two different time points in each group. There was no significant difference in the GAA levels at 0 h versus 24 h storage; between 0 h and 48 h; between 0 h and 72 h. However, when UCB samples were kept for 96 h, GAA levels were significantly altered and may not be reliable as shown in two separate groups of samples stored over this duration (Table 4). There was no difference in GAA levels if the DBS samples were dried <6 h or underwent 24 h/overnight drying (*p* = 0.282). We further proceeded with the equivalence testing using TOST method, which further confirmed the UCB group 0 h versus 24 h storage; 0 h versus 48 h storage, 0 h versus 72 h storage, as well as DBS drying < 6 h versus 24 h showed significant TOST with the upper and lower bound values set at 2 μM/h, −2 μM/h respectively, pointing towards equivalence.

### 3.6. Detection of a Novel Pathogenic Variant and Multiple Pseudodeficiency Alleles

Of the 4091 samples we screened, a total of 13 infants had GAA levels of ≤2.00 μM/h. We arbitrarily chose this value, which is slightly above the high-risk cut-off values for Pompe disease based on the NTUH (1.54 μM/h) and Burlina (1.89 μM/h) classifications. As the first wide-scale study of this population, analyses of cases with values slightly above the recommended cut-off levels for Pompe disease is intended to capture if any, pseudodeficiency cases previously reported among Asians that may appear in this group as well. From the report on GAA activity of Taiwanese newborn infants, pseudodeficiency alleles are recognized to attribute to GAA levels at the lower range of normal, thus shifting the population normal GAA distribution curve slightly to the left [23]. Similarly, in our study, 12 of the 13 infants with low GAA activity inherit various GAA pseudodeficiency alleles as shown in Table 5. The two most common pseudodeficiency alleles found in this study, appearing in at least half of the infants with low GAA levels, are c.1726G>A (p.Gly576Ser) Het. (*n* = 8/12), and c.2065G>A (p.Glu689Lys) Het. (*n* = 7/12). Almost all of these infants are of Malay ethnicity. The detailed distribution of alleles is shown in Table S3.

Of the 13 flagged samples with low GAA, one female infant (UKM-2617) had the lowest GAA, 0.38 μM/h. A repeat test for GAA activity on her peripheral venous blood sample shows an extremely low result of 0.53 μM/h. Subsequent genetic testing using the Sanger sequencing method (transcript NM_000152.5), identified two likely pathogenic variants (based on ACMG interpretation criteria) in the GAA gene, a novel heterozygous deletion variant, c.2005_2010del (p.Pro669_Phe670del) and a heterozygous missense variant, c.1123C>T (p.Arg375Cys). We conducted a parental segregation study to assess if the compound heterozygous variants are inherited ‘’in-trans’’ or ‘’in-cis’’, to aid in confirming the genetic diagnosis. The results show that the mother is heterozygous for c.2005_2010del (p.Pro669_Phe670del) and the father is heterozygous for c.1123C>T (p.Arg375Cys) (Table 6) which is consistent with a diagnosis of Pompe disease. A comprehensive clinical assessment was performed on this infant at the age of 3 months that showed appropriate growth parameters and developmental milestones with no apparent signs of neurological impairment. The echocardiogram examination revealed normal cardiac anatomy, with no structural abnormalities or signs of cardiac dysfunction, which suggested the clinical condition to be more likely a late-onset Pompe disease (LOPD) variant rather than IOPD.

## 4. Discussion

Pompe disease is the result of an inborn error of metabolism, also known as glycogen storage disease type II. Pathogenic variants in the GAA (alpha-1,4-glucosidase or acid maltase) gene causes partial or total deficiency of GAA enzyme, which leads to accumulation of lysosomal glycogen [4,7], forming storage vacuoles that are increased in numbers and size [24]. The estimated prevalence of Pompe disease varies, with higher rates reported in different populations of specific ethnic groups and geographic locations such as in Taiwan [24]. The pilot NBS programme in Taiwan showed that early detection at birth not only enables prompt treatment of IOPD patients, but it also makes it possible to identify people with asymptomatic GAA deficiency and underdiagnosed LOPD [25]. ERT with recombinant GAA (rhGAA) is the primary treatment for Pompe disease. Screening tests involve measuring the GAA enzyme levels in peripheral blood, where individuals with Pompe disease typically show very low GAA enzyme activity, less than 1% of normal levels. Early diagnosis through NBS has been implemented in many high-income countries [11]. NBS in developing countries in the South-East Asian and North African regions are limited [26]. An NBS program is essential to facilitate the timely initiation of ERT. Early intervention can prevent progressive muscle damage and significantly improve prognosis.

NBS is recognised as a vital preventive health programme for early detection and treatment of inherited metabolic conditions before disease manifestation [27]. Most assays are based on the analysis of enzyme activities against artificial standards incorporated onto the DBS, followed by analysis either by fluorometry, tandem mass spectrometry, or microfluidics combined with fluorometry [28]. In 2003, a fluorometric assay was created using 4-methylumbelliferyl-α-d-glucopyranoside (4-MUG) as the substrate that could differentiate DBS of patients with IOPD from obligate heterozygotes and healthy individuals [29].

Several previous studies have compared the usage of UCB and peripheral blood for NBS [30], with UCB less than 72 h validated favorably to screen for congenital hypothyroidism [31,32]. The UCB samples can also be used for hemoglobinopathies screening [33], while another study reported screening collectively for congenital hypothyroidism, cystic fibrosis, G6PD deficiency, and profound biotinidase deficiency [34]. The UCB method offers several advantages, mainly in terms of logistics, whereby test results and confirmatory samples can be organised quickly before infants are discharged early [33,35]. Furthermore, African doctors have observed that families are more receptive to cord blood collection than heel-pricks [33]. Screening with UCB may also be associated with a lower recall rate and fewer false positive readings than heel-prick samples besides being inexpensive and readily available [36,37].

Our study validated the use of UCB on DBS for screening of Pompe disease and showed that the average GAA in UCB is similar to the Day 3 heel-prick blood samples from Taiwanese newborn infants. The cut-off value of GAA (<1.54 μM/h) based on the 0.1% of the Taiwanese population average was sensitive to detect one patient who has Pompe disease out of just over 4000 UCB samples screened. The NTUH cut-off value for high risk Pompe disease set at 0.1% of the population average for GAA, is more pragmatic in our setting in view of the significant proportion of non-pathogenic variants with lower GAA values. If the Burlina classification was used (<1.89 μM/h), there may be a significantly higher number of flagged samples and call-back for re-testing, which may utilize more resources for contact tracing and creating undue parental anxiety. In the context of our study, an additional six cases would have been called back for repeat testing. In accordance with previous reports, this study also confirmed a significant proportion of infants with non-Pompe disease, inheriting non-pathogenic pseudodeficient genetic variants [23,38]. Adopting the Taiwan experience, Pompe disease is characteristically associated with a GAA level much lower than 1 μM/h, which supports the choice of a lower GAA cut-off value in screening for Pompe disease in our population.

We found that female infants have significantly higher mean GAA levels, which we do not yet know the clinical significance. Of interest, the Missouri newborn screening program in reviewing their dataset of 302,328 samples showed mean GAA activity for females was also higher than males [39]. Mean GAA levels are also significantly lower in infants who are VLBW, which is also of undetermined clinical significance. The GAA levels are not affected by different ethnic backgrounds, with predominantly Malay rather than Chinese ancestry. Importantly, we found that the UCB GAA levels are not equivalent to Day 1 or Day 2 peripheral blood or heel-prick samples. The average GAA level is about 30% higher in Day 1 peripheral blood, 15% more in Day 2, than UCB. This may be due to the physiological surge in total leukocytes in the peripheral blood in the first 6–24 h in response to the stress and transitioning after birth [40]. From this study, we have derived a formula to extrapolate the GAA enzyme level of Day 1 peripheral blood sample to UCB. This is of practical importance if the UCB sampling were missed, insufficient or unsatisfactorily blotted.

The bi-allelic genetic alterations in the GAA gene leading to Pompe disease in our patient has not been documented in the scientific literature or public genetic databases. The c.2005_2010del (p.Pro669_Phe670del) is a deletion variation in the GAA gene where a deletion of six nucleotides occurs at positions 2005 to 2010. This deletion leads to the loss of two amino acids, Proline (Pro) at position 669 and Phenylalanine (Phe) at position 670. The maximum minor allele frequency of this variant across the human population database is 0.0000% and it has not been reported in ClinVar. The c.1123C>T variant is a missense variant which refers to a single nucleotide change at position 1123, where a cytosine (C) is replaced by a thymine (T). The maximum minor allele frequency of this variant across the human population database is 0.0500%. Based on ACMG interpretation criteria, this variant is classified as likely pathogenic; however, it is listed in ClinVar with conflicting interpretations of pathogenicity at the time of reporting. Thirteen in silico prediction tools suggest this variant has a deleterious effect on protein structure and function. Additionally, based on an in vitro expression system, Goomber et al. showed that c.1123C>T was associated with reduced GAA enzyme activity (11.7% of wild-type control). Protein characterization studies indicated this variant could impact on GAA protein stability and processing and it was finally classified as likely pathogenic [41]. In our case, clinical assessment of this affected infant during the first year of life showing absence of any concerning abnormalities of the musculature, delayed development or cardiac dysfunction suggests that this variant of the GAA gene most likely is associated with LOPD. However, continued monitoring and follow-up examinations are essential to detect any evolving changes or complications over time and to consider starting ERT if necessary.

During the 18-month study period, this single case confirmed to have Pompe disease out of 4091 newborn infants screened at our hospital, yields a prevalence of 0.024%, equivalent to 24 cases per 100,000 births. The population prevalence could plausibly range from 1 in 161,000 to 1 in 735, based on the 95% confidence interval. This observed frequency 1 in 4091 is notably higher than the published global incidence estimate of approximately 1 in 40,000 for Pompe disease overall [1]. Although our finding is based on a single case, it may reflect population-specific differences, a limited sample size, or other contributing factors, such as a more commonly occurring form of the disease, LOPD. Larger-scale nationwide and multicenter studies are warranted to determine whether this reflects a true increased prevalence in our population. A recent publication reported the severe form, IOPD, in this population a cohort of 17 cases inheriting 14 different and 3 novel mutations in the GAA gene with the most common being c.1935C>Ap.(D645E) [42].

This study also confirmed the presence of pseudodeficient variants, whereby 12 of these newborn infants have GAA levels of <2 μM/h and carrying 28 mutated alleles with 6 different variants. The 6 GAA variants are: c.1062C>G, c.1726G>A, c.2065G>A, c.1987C>T, c.913G>A, and c.841C>T. The most common variants are c.1726G>A and c.2065G>A, occurring in all 12 newborns, with allele frequency of 42.9% each. These two variants are also known as Asian c.[1726G>A; 2065G>A] pseudodeficiency [23] and are missense. Out of the 28 alleles analyzed, 26 are missense and 2 are nonsense. The variants c.1062C>G, c.1726G>A, c.2065G>A, and c.841C>T are identified through Pompe disease GAA variant database (http://www.pompevariantdatabase.nl) (accessed on 12 December 2023), while variants c.1987C>T and c.913G>A were identified through ClinVar (https://www.ncbi.nlm.nih.gov/clinvar/) (accessed on 12 December 2023). Overall, for Pompe disease, it may be challenging to find the correlation between genotype and phenotype because of the diverse genetic variants. Up to 20% of the variants reported in the GAA variant database are described without a strict correlation of genotype–phenotype. This argues for a primary enzyme-based NBS, while a genetic-based NBS for Pompe disease may still need to be accompanied by enzyme activity testing for confirmatory diagnosis.

The use of UCB for NBS has several limitations. The detection of disorders based on metabolite accumulation may be impaired by inadequate loading into the system when dietary intake is not yet established. The current global standard of practice to delay clamping of the umbilical cord at birth should be studied in the context of utilizing UCB for NBS with regards to the sufficiency and quality of the UCB sample, although intuitively this should not pose as a significant impediment since the sample volume required is not much. Conversely, parental choices to bank their newborn UCB need to be addressed accordingly as the harvesting of UCB for this purpose is more intricate and restrictive to other related interventions. Lastly, obtaining a good quality UCB sample and blotting it to make DBS needs skills to be acquired from appropriate training of the healthcare staff involved in this procedure. For the success and sustainability of an NBS program, it will need education programs to increase awareness to both caregivers and recipients, and stakeholder buy-in on how this screening process could improve health outcomes of all infants.

## Figures and Tables

**Table 1:** The GAA values in UCB samples.

			Cord Blood GAA (μM/h)
	Dataset A (Total Original)	Dataset B (Excluded Samples of Suspect Quality)	Dataset C (Excluded Outlier Values)
*n*	4091	3987	3980
Mean (SD)	10.04 (5.95)	10.08 (5.98)	9.96 (4.05)
Median (IQR)	9.43 (5.25)	9.47 (5.21)	9.46 (5.20)
Skewness (SE)	19.50 (0.04)	19.66 (0.04)	0.76 (0.04)
Kurtosis (SE)	747.50 (0.08)	750.30 (0.08)	0.81 (0.08)

**Table 2:** Reference cut-off values of acid alpha-glucosidase (GAA) enzyme in umbilical cord blood for the diagnosis of Pompe disease [22].

Values	Dataset A (*n* = 4091)	Dataset B (*n* = 3987)	Dataset C (*n* = 3980)
Burlina et al. classification:			
-Normal	>2.36	>2.37	>2.37
-Borderline risk	1.89–2.36	1.89–2.37	1.89–2.37
-High risk	<1.89	<1.89	<1.89
NTUH classification:			
-High possibility of Pompe disease	≤1.54	≤1.54	≤1.54

**Table 3:** Comparison of GAA levels in paired UCB and Day 1 peripheral blood samples.

		*n*	Mean GAA, μM/h (SD)	*p*-Value
Dataset A (complete paired samples)	Cord Blood	103	8.62 (4.93) ^	<0.001
	Peripheral Blood	103	13.21 (9.56) ^	
Dataset B (excluded poor quality samples)	Cord Blood	97	8.69 (4.81) ^	<0.001
	Peripheral Blood	97	13.23 (9.46) ^	
Dataset C (excluded outlier values)	Cord Blood	96	9.37 (3.97)	<0.001
	Peripheral Blood	96	13.79 (6.10)	

Note: ^—median (interquartile range).

**Table 4:** Effects of UCB sample storage before blotting to DBS card and sample drying time on GAA levels.

Subgroups	Time (h)	*n*	GAA Mean (SD) [μM/h]	*p*-Value *
Samples kept at 4 °C				
0-h vs. 24-h	0 24	30 30	9.89 (3.16) 10.05 (3.03)	0.563
0-h vs. 48-h	0 48	30 30	11.32 (5.23) 11.26 (5.25)	0.804
0-h vs. 72-h	0 72	30 30	8.05 (4.20) ^ 9.46 (5.30) ^	0.064 ^^
0-h vs. 96-h	0 96	30 30	9.22 (4.21) ^ 10.05 (4.55) ^	0.035 ^^
48-h vs. 96-h	48 96	30 30	9.83 (5.35) ^ 9.01 (5.21) ^	0.006 ^^
Drying of DBS				
<6-h vs. 24-h	<6-h 24-h	30 30	9.88 (3.15) 9.69 (3.17)	0.282

* *p*-value of paired *t*-test unless otherwise specified; ^—median (interquartile range); ^^—Wilcoxon signed-rank test.

**Table 5:** GAA pseudodeficiency alleles and low GAA levels in UCB.

No	ID	UCB GAA (μM/h)	GAA Pseudodeficiency Alleles *
1	UKM-3006	0.85	c.1726G>A (p.Gly576Ser) Hom. c.2065G>A (p.Glu689Lys) Hom.
2	UKM-3108	1.47	c.841C>T(p.Arg281Trp) Het. c.1726G>A (p.Gly576Ser) Het. c.2065G>A (p.Glu689Lys) Het.
3	UKM-0413	1.54	c.1987C>T (p.Gln663Ter) Het. c.1726G>A (p.Gly576Ser) Het. c.2065G>A (p.Glu689Lys) Hom.
4	UKM-3917	1.55	c.1726G>A (p.Gly576Ser) Het. c.2065G>A (p.Glu689Lys) Het.
5	UKM-0154	1.57	c.1062C>G (p.Tyr354Ter) Het. c.1726G>A (p.Gly576Ser) Het. c.2065G>A (p.Glu689Lys) Het.
6	UKM-3649	1.62	c.1726G>A (p.Gly576Ser) Het. c.2065G>A (p.Glu689Lys) Het.
7	UKM-3383	1.68	c.1726G>A (p.Gly576Ser) Het. c.2065G>A (p.Glu689Lys) Het.
8	UKM-2219	1.74	c.913G>A(p.Gly305Arg) Het. c.1726G>A (p.Gly576Ser) Het. c.2065G>A (p.Glu689Lys) Het.
9	UKM-1334	1.83	c.1726G>A (p.Gly576Ser) Hom. c.2065G>A (p.Glu689Lys) Hom.
10	UKM-2501	1.89	c.1726G>A (p.Gly576Ser) Hom. c.2065G>A (p.Glu689Lys) Hom.
11	UKM-0812	1.91	c.1726G>A (p.Gly576Ser) Het. c.2065G>A (p.Glu689Lys) Het.
12	UKM-2791	2.00	c.1726G>A (p.Gly576Ser) Hom. c.2065G>A (p.Glu689Lys) Hom.

* Sanger sequencing of the GAA gene using reference transcript NM_000152.5.

**Table 6:** Novel pathogenic variant of the GAA gene in a female infant with extremely low UCB GAA level.

No	ID	GAA Level (μM/h)	GAA (Likely) Pathogenic Alleles *	Interpretation
1	UKM-2617	0.38 (from UCB) 0.53 (Repeat, from venous blood sample)	c.1123C>T (p.Arg375Cys) Het. c.2005_2010del (p.Pro669_Phe670del) Het.	Pompe disease
2	Father	2.50	c.1123C>T (p.Arg375Cys) Het.	Pompe disease carrier
3	Mother	2.90	c.2005_2010del (p.Pro669_Phe670del) Het.	Pompe disease carrier

* Sanger sequencing of the GAA gene using reference transcript NM_000152.5.

## Data Availability

Data of this study are available upon reasonable request.

## Supplementary Materials

The following supporting information can be downloaded at: https://www.mdpi.com/article/10.3390/ijns11030074/s1, Figure S1: UCB samples obtained for GAA measurement; Figure S2: Histogram for cord blood GAA levels (µM/h): (a) Dataset A (*n* = 4091); (b) Dataset B (*n* = 3987); (c) Dataset C (*n* = 3980); Figure S3: Collection of paired UCB and Day 1 peripheral venous blood samples for GAA levels; Table S1: Distribution of normal and at-risk infants for Pompe disease based on the cut-off GAA levels of Burlina et al. and NTUH classifications; Table S2: Comparison of cord blood GAA values between gender, maternal ethnicity, and birth weight categories; Table S3: The frequency of pseudodeficiency alleles in the GAA gene and demographic characteristics of newborn infants (*n* = 12) with low UCB GAA levels (≤2.00 μM/h).

## Abbreviations

The following abbreviations are used in this manuscript:

GAA	Acid Alpha-Glucosidase
IOPD	Infantile-Onset Pompe Disease
ERT	Enzyme Replacement Therapy
NBS	Newborn Screening
DBS	Dried Blood Spots
UCB	Umbilical Cord Blood
ICC	Intraclass Correlation Coefficient
NTUH	National Taiwan University Hospital
LSD	Lysosomal Storage Disease
HGMD	Human Gene Mutation Database
HCTM	Hospital Canselor Tuanku Muhriz
LBW	Low Birth Weight
VLBW	Very Low Birth Weight
ELBW	Extremely Low Birth Weight
TOST	Two-One-Sided-Test
LOPD	Late Onset Pompe Disease
