# Supplementary material for: Umbilical Cord Blood Sampling for Newborn Screening of Pompe Disease and the Detection of a Novel Pathogenic Variant and Pseudodeficiency Variants in an Asian Population
Source: Int J Neonatal Screen. 2025 Sep 3;11(3):74. doi: 10.3390/ijns11030074 (PMC12452700; doi:10.3390/ijns11030074)
Supplement: Supplementary file 1 [file IJNS-11-00074-s001.zip › IJNS-3778529-supplementary.pdf]

Supplementary Materials

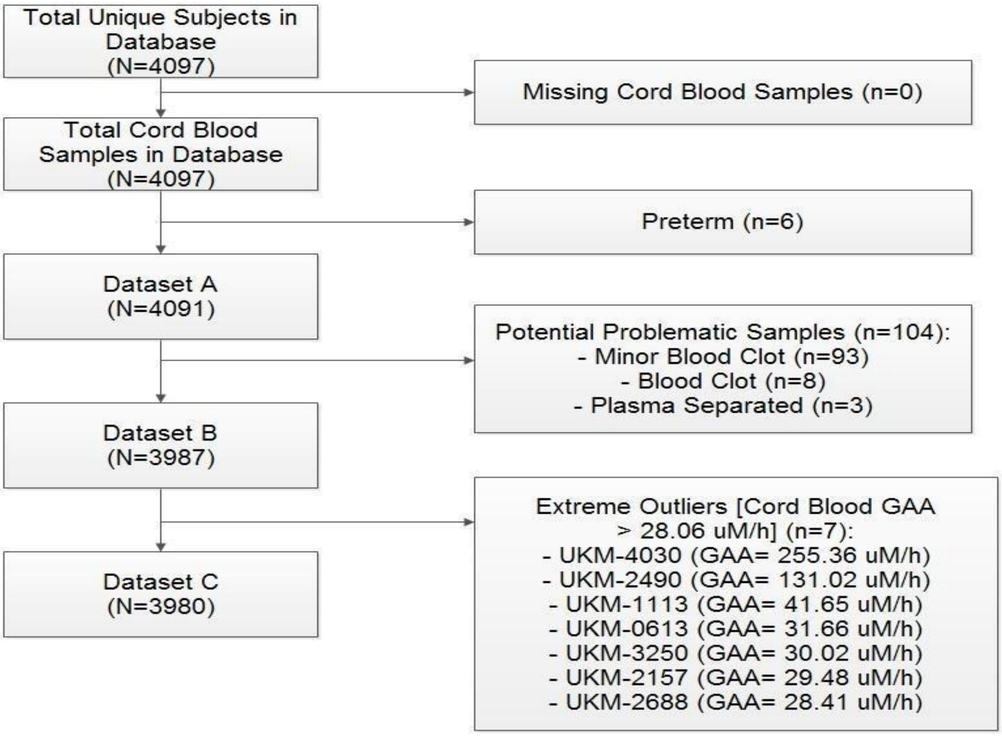

Figure S1: UCB samples obtained for GAA measurement

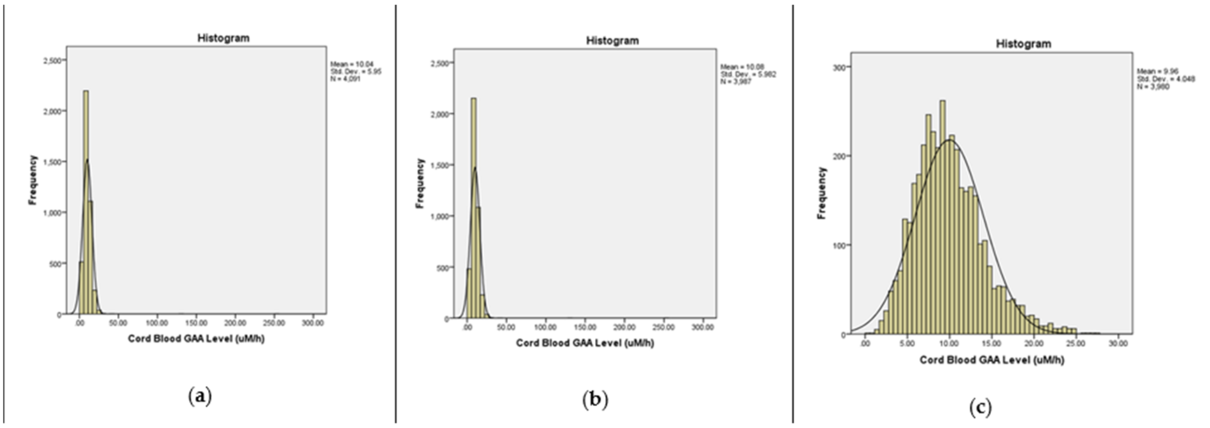

Figure S2: Histogram for Cord Blood GAA Levels (μM/h): (a) Dataset A (n=4091); (b) Dataset B (n=3987); (c) Dataset C (n=3980)

	Dataset A		Dataset B		Dataset C	
	n	%	n	%	n	%
Cord Blood GAA Classification (Based on Burlina et al.)						
Normal	4060	99.25	3959	99.30	3952	99.30
Borderline	21	0.50	18	0.45	18	0.45
High Risk	10	0.25	10	0.25	10	0.25
Cord Blood GAA Classification (Based on NTUH)						
Low Possibility of Pompe Disease	4087	99.9	3983	99.9	3976	99.9
High Possibility of Pompe Disease	4	0.1	4	0.1	4	0.1

Table S1: Distribution of normal and at-risk infants for Pompe disease based on the cut-off GAA levels of Burlina et al. and NTUH classifications

Source: Cheah, F.C., et al. 2025. Reference cut-off values of acid alpha-glucosidase (GAA) enzyme in umbilical cord blood for the diagnosis of Pompe disease. Universiti Kebangsaan Malaysia, 2025; Bangi, Malaysia.

		Dataset A (n=4091)		Dataset B (n=3987)		Dataset C (n=3980)	
		**n (%)**	**GAA Mean (SD) [uM/h]**	**n (%)**	**GAA Mean (SD) [uM/h]**	**n (%)**	**GAA Mean (SD) [uM/h]**
Gender							
Male		2085 (50.9)	9.08 (4.97)#	2036 (51.0)#	9.12 (4.91)#	2032 (51.0)	9.61 (3.98)
Female		2005 (49.0)	9.83 (5.45)#	1950 (48.9)#	9.83 (5.36)#	1947 (48.9)	10.32 (4.09)
Ambiguous		1 (<0.1)	(Excluded from analysis)	1 (<0.1)	(Excluded from analysis)	1 (<0.1)	(Excluded from analysis)
p-value (Student's t-test)			<0.001* ^		<0.001* ^		<0.001*
Maternal Ethnicity							
Malay	Unweighted	3435 (84.0)	9.46 (5.23)#	3345 (83.9)	9.45 (5.21)#	3338 (83.9)	9.99 (4.06)

	Weighted	236 (57.4)[$]	10.08 (0.11)[$$]	2275 (57.1)[$]	10.13 (0.11)[$$]	2270 (57.1)[$]	9.99 (0.07)[$$]
Chinese	Unweighted	420 (10.3)	9.56 (5.43)[#]	410 (10.3)	9.56 (5.43)[#]	410 (10.3)	9.97 (4.21)
	Weighted	937 (23.0)[$]	9.90 (0.21)[$$]	914 (23.0)[$]	9.97 (0.21)[$$]	914 (23.0)[$]	9.97 (0.21)[$$]
Indian	Unweighted	123 (3.0)	9.10 (4.40)[#]	120 (3.0)	9.10 (4.40)[#]	120 (3.0)	9.49 (3.51)
	Weighted	274 (6.7)[$]	9.54 (0.32)[$$]	268 (6.7)[$]	9.49 (0.32)[$$]	268 (6.7)[$]	9.49 (0.32)[$$]
Non-Malay indigenous	Unweighted	63 (1.5)	9.16 (5.50)[#]	63 (1.6)	9.16 (5.50)[#]	63 (1.6)	9.65 (4.04)
	Weighted	496 (12.2)[$]	9.65 (0.50)[$$]	496 (12.5)[$]	9.65 (0.50)[$$]	496 (12.5)[$]	9.65 (0.50)[$$]
Others	Unweighted	50 (1.2)	9.54 (3.83)[#]	49 (1.2)	9.54 (3.83)[#]	49 (1.2)	9.54 (3.45)
	Weighted	29 (0.7)[$]	9.76 (0.52)[$$]	28 (0.7)[$]	9.54 (0.49)[$$]	28 (0.7)[$]	9.54 (0.49)[$$]
p-value (One-way ANOVA)	*Unweighted*	0.834^^		0.712^^		0.617	
p-value (Weighted GLM)	*Weighted*	0.481		0.256		0.491	
Birth Weight							
Macrosomia (>4.00 kg)		59 (1.4)	9.72 (5.40)[#]	58 (1.5)	9.81 (5.49)[#]	58 (1.5)	10.32 (4.28)
Normal (2.50 kg to 4.00 kg)		3713 (90.8)	9.49 (5.24)[#]	3624 (90.9)	9.54 (5.21)[#]	3619 (91.0)	10.02 (4.06)
Low birth weight (>1.50 kg & =<2.50 kg)		312 (7.6)	8.59 (4.79)[#]	299 (7.5)	8.66 (4.61)[#]	297 (7.5)	9.16 (3.79)
Very low birth weight (>1.00 kg & =<1.50 kg)		2 (<0.1)	5.89 (Not valid)[#]	2 (0.1)	5.89 (Not valid)[#]	2 (0.1)	5.89 (2.66)
Extremely low birth weight (=<1.00 kg)		3 (0.1)	11.33 (Not valid)[#]	3 (0.1)	11.33 (Not valid)[#]	3 (0.1)	12.73 (2.85)
Missing data		2 (Excluded from analysis)	(Excluded from analysis)	1 (Excluded from analysis)	(Excluded from analysis)	1 (Excluded from analysis)	(Excluded from analysis)
p-value (One-way ANOVA)		0.001* ^^		.001* ^^		0.002* [@]	
Pearson's r [Birth weight as continuous variable versus GAA]		0.078 ^^^		0.073 ^^^		0.066	
R^2		0.006 ^^^		0.005 ^^^		0.004	
p-value		<0.001* ^^^		<0.001* ^^^		<0.001*	

Note: [#] - Median (Interquartile Range); [$] - Estimated n (%); [$$] - Estimated Mean; ^ - Mann-Whitney's Test; ^^ - *Kruskal-Wallis Test;* ^^^ *Spearman's rho;* * - p<0.05 (statistical significance)

@ - Posthoc test (Bonferroni) showed the significant difference was mainly between the normal and LBW categories

Table S2: Comparison of cord blood GAA values between gender, maternal ethnicity and birth weight categories

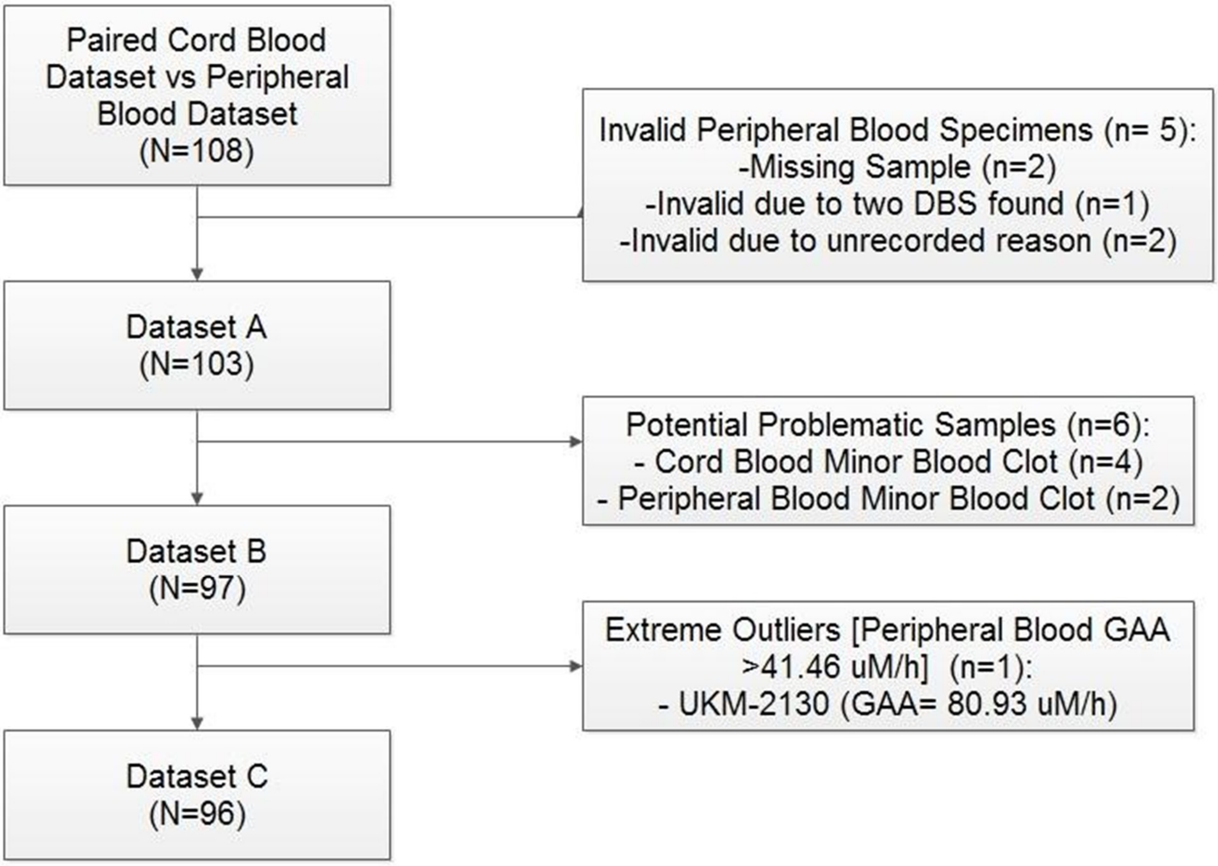

Figure S3: Collection of paired UCB and day 1 peripheral venous blood samples for GAA levels.

NM_000152.5(GAA):	n (%)	
c.1726G>A (p.Gly576Ser) Het.	8 (67)	
c.2065G>A (p.Glu689Lys) Het.	7 (58)	
c.2065G>A (p.Glu689Lys) Hom.	5 (42)	
c.1726G>A (p.Gly576Ser) Hom.	4 (33)	
c.1062C>G (p.Tyr354Ter) Het.	1 (8)	
c.1987C>T (p.Gln663Ter) Het.	1 (8)	
c.841C>T(p.Arg281Trp) Het.	1 (8)	
c.913G>A(p.Gly305Arg) Het.	1 (8)	
	n	%
Sex		
Male	6	50.0
Female	6	50.0
Ethnicity		
Malay	1	91.7
Chinese	1	8.3
Birth Weight		
Normal (2.50 kg to 4.00 kg)	1	91.7
LBW (>1.50 kg & =<2.50 kg)	1	8.3

Table S3: The frequency of pseudodeficiency alleles in the GAA gene and demographic characteristics of newborn infants (n=12) with low UCB GAA levels (≤2.00 μM/h).
